# Radiographic evaluation of bone regeneration after the application of plasma rich in growth factors in a lower third molar socket: a case report

**DOI:** 10.1186/1757-1626-2-9134

**Published:** 2009-12-03

**Authors:** Ioannis Nazaroglou, Christos Stavrianos, Panagiotis Kafas, Euthimios Matoulas, Tahwinder Upile, Irodis Barlas, Waseem Jerjes

**Affiliations:** 1 Department of Oral Surgery, Surgical Implantology and Radiology, School of Dentistry, Aristotle University, Agiou Dimitriou Street, Thessalonica, 541 24, Greece; 2 Department of Endodontics, School of Dentistry, Aristotle University, Agiou Dimitriou Street, Thessalonica, 541 24, Greece; 3 Department of Surgery, University College London Medical School, Gower Street, London, WC1E 6BT, UK; 4 UCLH Head and Neck Centre, Unit of Oral and Maxillofacial Surgery, UCL Eastman Dental Institute, 256 Gray's Inn Road, London, WC1X 8LD, UK

## Abstract

A 42-year-old Mediterranean male presented complaining of inability to sustain good oral care at the posterior aspect of the lower right jaw. The main problems were food impaction in the area and the subsequent malodor. The patient reported remarkable medical history. Clinical examination revealed local erytherma with noticeable bone defect distal to the second molar with obvious defect in the mesial wall of the third molar; the penetration depth was found to be up to 6 mm.

Radiological evaluation confirmed the defect and it was attributed to the mesioangularly partially impacted lower third molar. It was decided that third molar should be extracted and concentrate of the patient's growth factors (PRGF) to be applied into the bony defect to stimulate bone regeneration and promote healing.

The third molar tooth was, then, removed surgically and the PRGF, which was prepared preoperatively, was implanted in the socket. At the first postoperative day, moderate pain was the main complaint and was controlled by NSAIDs. One week postoperatively, the sutures were removed and there was good tissue healing on examination.

On the fiftieth postoperative day, radiographic evaluation took place and showed noticeable enhancement of density and radio-opacity in the third molar socket area, in comparison with the baseline image. Further, clinical examination showed significant reduction of periodontal pocketing and evidence of new bone formation.

In conclusion, PRGF was very successful in stimulating bone regeneration and promote healing following dental extraction.

## Background

Being the last teeth to erupt in the jaw and due to the lack of space, third molars tend to fail to achieve their normal position in the jaw. As a result, they become impacted (failure to erupt into the normal functional position), partially or completely. Patients report pain, recurrent swelling and/or infection which most often fail to respond to conventional medical management. Thus surgical removal of the problematic tooth is quite common, this may involve surgical exposure, removal of surrounding bone and tooth sectioning [1].

More problems arise if the impacted teeth are malinclined and in contact with adjacent teeth. This usually leads to food entrapment, bacterial colonization and eventually damage to adjacent dental and bony structures. Initially, this can be manifested by pain, swelling, inflammation, malodor and end up by abscess formation and dental mobility (most commonly reported in the second molar tooth) [1]. The only available option here is surgical extraction of the third molar followed by debridemnt and curettage of granulation tissue and debris in the area.

It has been hypothesized that the immediate application of autogenous growth factors into defective bony areas enhances soft tissue healing and stimulate bone regeneration [2]. In this case study, we report our findings following the application of plasma-rich in growth factors (PRGF) to defective bony socket immediately after surgical extraction of the lower third molar.

## Case presentation

**Figure 1:**
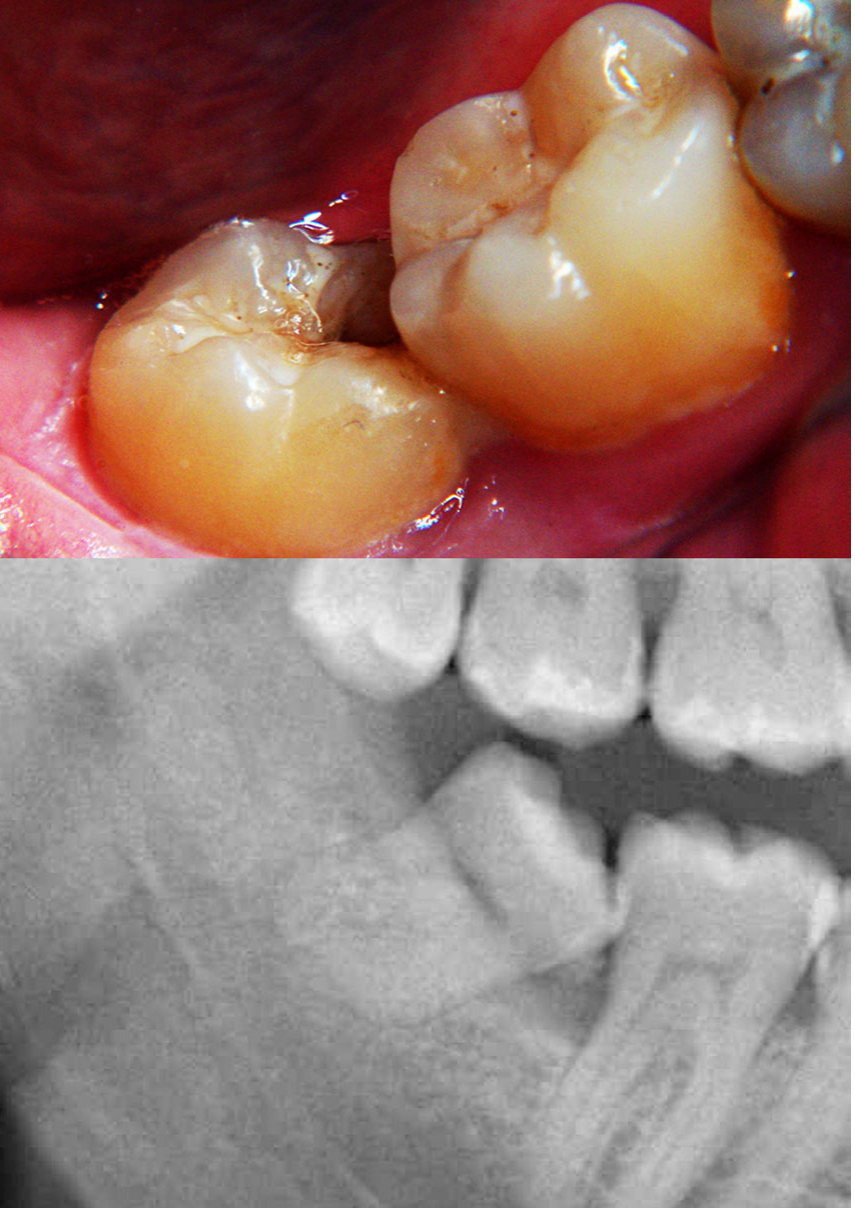
**(a) Intraoral view showing defect in the mesial wall of the third molar associated with bony defect distal to the second molar and local erythema**. (b) Preoperative radiographic image showing mesially impacted third molar in contact with the distal surface of the second molar causing radiolucency at the contact point (dental decay). There is also slight periradicular radiolucency (inflammation) and dilation or thickening of the lamina dura.

A 42-year-old Mediterranean male presented complaining of inability to sustain good oral care at the posterior aspect of the lower right jaw. The main problems were food impaction in the area and the subsequent malodor. The patient reported remarkable medical history, and he was a non-smoker. Clinical examination revealed local erytherma with noticeable bony defect distal to the second molar with obvious defect in the mesial wall of the third molar; the penetration depth was found to be up to 6 mm (Figure 1a).

Radiological evaluation confirmed the defect and it was attributed to the mesioangularly impacted lower third molar; there was marked dilation or thickening of the lamina dura secondary to the local inflammatory reaction. It was decided that the third molar should be extracted and concentrate of the patient's growth factors (PRGF) to be implanted into the bony defect to stimulate bone regeneration and promote healing(Figure 1b).

**Figure 2:**
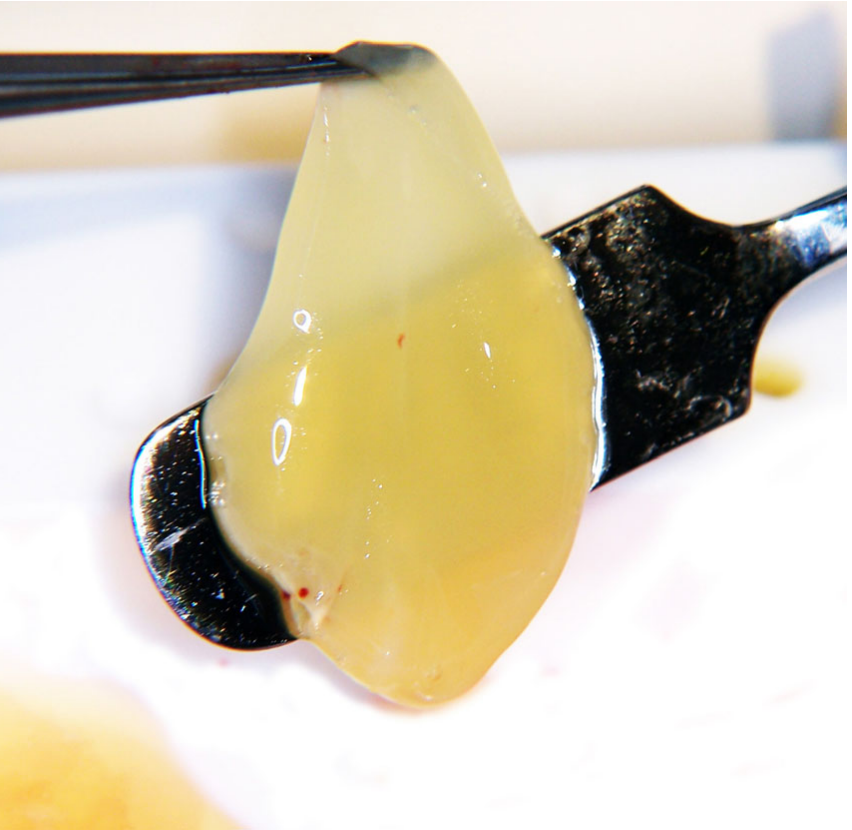
**The scaffold-like PRGF after activation with CaCl_2 _is mounted on the spatula before implantation in the bony defect**.

Preoperatively, 24 cc of the patient's blood (venous blood from a peripheral vessel) was obtained using a butterfly cannula. The blood was collected in five sterile glass tubes, pretreated with 3,8% trisodium citrate (anticoagulant factor) and then centrifuged at 460 g for 8 mins at room temperature (PRGF System, BTI Biotechnology Institute, Vitoria, Spain) [3]. After centrifugation, blood was separated into distinct layers, with the cellular fraction located at the bottom of the tubes and the plasmatic fraction located just above the red blood cell line. Plasma volume constituted 1 cc and was located just above the red blood cell line. This fraction appears to be very rich in growth factors [4]. A volume of approximately 5 cc of PRGF was collected in a tube and 50 μl of 10% calcium chloride (CaCl_2_) were added per 1 cc of PRGF [4]. CaCl_2 _activates PRGF and stimulates the formation of a semi-solid, scaffold-like mass which functions as a matrix for progenitor cells and maintains the regenerative area of the defect [5]. After activation, PRGF was mounted on a spatula and ready to be applied to the bony defect (Figure 2).

**Figure 3:**
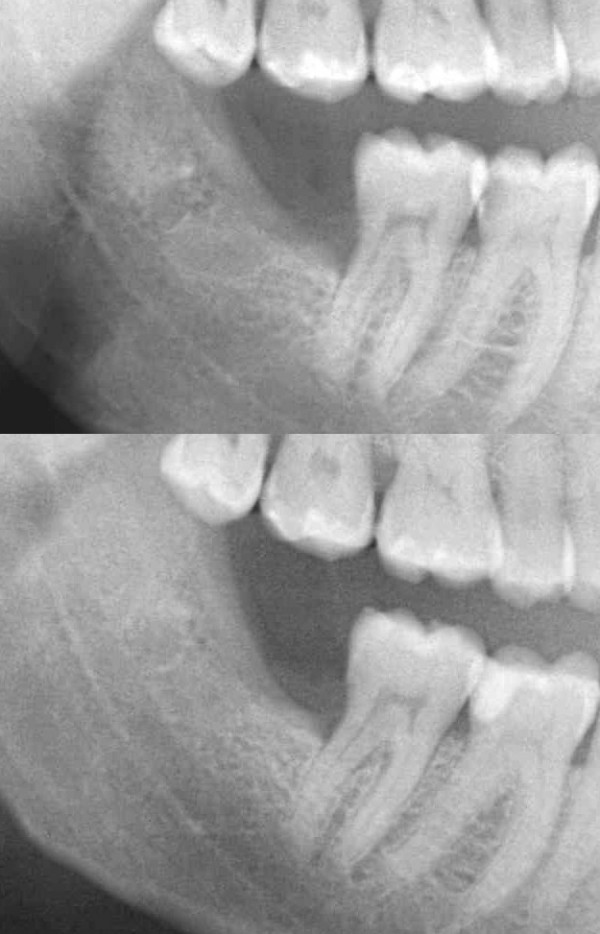
**(a) An immediate postoperative dental panoramic tomography was obtained to be considered as a baseline image for this case study**. (b) Radiographic image, obtained 50 days postoperatively showing enhancement of radiopacity in the third molar socket area.

The surgical part of this case study took place under local anesthesia. A transginigival incision was made after tissue infiltration with local anesthesia. A buccal mucoperiosteal flap was raised and bone was exposed. The third molar was elevated and removed, followed by debridement and curettage of all debris and granulation tissue in the area. Subsequently, the 'scaffold-like', CaCl_2 _activated PRGF was implanted in the bony defect. The volume of PRGF was adequate to provide full cover of the whole defect. The flap was carefully repositioned and sutured with horizontal mattress sutures. An immediate postoperative dental panoramic tomography was obtained and considered as the "baseline image" for this case study (Figure 3A). The patient was given full postoperative instructions, including contact details if postoperative complications to occur. Also, anti-inflammatory and antimicrobial cover was provided for 5 days.

At the first postoperative day, moderate pain was the main complaint and was controlled by NSAIDs. After two days, the pain subsided. No postoperative swelling was reported by the patient. There were neither symptoms nor clinical suspicion that would suggest alveolar osteitis (dry socket), indicating normal clotting and coagulation [1,2]. One week postoperatively, the sutures were removed and there was good tissue healing on examination.

On the fiftieth postoperative day, and according to the PRGF clinical protocol, radiographic evaluation took place and showed noticeable enhancement of density and radio-opacity in the third molar socket area, in comparison with the baseline image (Figure 3a and Figure 3b). Further clinical examination showed significant reduction of periodontal pocketing by 3 mm and evidence of new bone formation.

## Conclusion

It is widely known that platelets, including multiple growth factors, play a "key role" in tissue regeneration. For example, platelet-derived growth factor (PDGF) acts as an induction system for connective tissue cells, vascular endothelial growth factor (VEGF) enhances endothelial cell proliferation and migration and consequently provokes angiogenesis; while transforming growth factor-β (TGF - β) motivates osteoprogenitor cells to proliferate and insulin-like growth factor(IGF-I) stimulates the differentiation and productivity of osteoblasts [5].

PRGF consist of a minute volume of plasma, with high concentration of platelets (including growth factors) [5]. It is can be acquired after centrifugation of the patient's blood. Compared to other formulations, PRGF has two major differences; the growth factors-rich fraction is activated by exogenous calcium chloride instead of thrombin, enabling increase of the physiological release of growth factors [6]. Also, the final product is leukocytes-depleted, to avoid the pro-inflammatory action of proteases and acid hydrolases [7]. Hence, PRGF can be described as having an "optimal regenerative potential".

Several authorities confirmed the positive effect of autogenous human growth factors on hard tissue regeneration. In these studies, high concentrations of growth factors were applied either as platelet-rich plasma (PRP) or as plasma-rich in growth factors (PRGF) [1-5,8-10]. Other research studies reported that platelet-rich products may accelerate bone regeneration and consequently shorten healing time [8,9]. More specifically, bony defects in the oral cavity were found to respond positively to PRP which leads to accelerated bone formation with confirmatory radiological changes in one month postoperatively [2].

In the current case study, we applied PRGF and showed a noticeable enhancement of radiographic density, just 50 days after the operation. The PRGF was placed in the extraction socket (bony defect) with the aim to preserve the bony quantity, treat the periodontal pocket, improve bone vertical dimension for future implant placement and to promote soft tissue healing. By using the patients own blood to generate the growth factor, we avoid any possible immune or biocompatibility problems that can occur when using synthetics or heterologous grafting materials [11].

Based on this empirical evidence, we suggest that PRGF application in alveolar defects may have a positive outcome when it comes to bone regeneration and soft tissue healing, especially when the area is compromised with local inflammation/infection. Further studies are required to establish the effect of PRGF in the preservation of bone following surgery. The need for a double-blind randomized controlled trial should be emphasized in order to evaluate the efficacy of this technique.

## Abbreviations

PRGF: plasma-rich in growth factors; CaCl_2_: calcium chloride; PDGF: platelet-derived growth factor; VEGF: vascular endothelial growth factor; TGF-β: transforming growth factor-β; IGF-I: insulin-like growth factor I; PRP: platelet-rich plasma.

## Consent

Written informed consent was obtained from the patient for publication of this case report and accompanying images. A copy of the written consent is available for review by the Editor-in-Chief of this journal.

## Competing interests

The authors declare that they have no competing interests.

## Authors' contributions

IN examined, diagnosed, treated and followed up the patient. IN, CS, PK, EM, TU, IB and WJ were responsible for conception and design and acquisition of data. IN wrote the initial draft. PK, WJ and TU helped revise the manuscript. IN obtained consent from the patient. All authors approved the final manuscript.
